# Impact of Physical Exercise on Breast Cancer-Related Lymphedema and Non-Invasive Measurement Tools: A Systematic Review

**DOI:** 10.3390/cancers17020333

**Published:** 2025-01-20

**Authors:** Marta Arias-Crespo, Rubén García-Fernández, Natalia Calvo-Ayuso, Cristian Martín-Vázquez, Maria de Fátima da Silva Vieira Martins, Enedina Quiroga-Sánchez

**Affiliations:** 1 Department of Nursing and Physiotherapy, Campus de Ponferrada, Universidad de León, 24401 León, Spain; mariac@unileon.es (M.A.-C.); cmartv@unileon.es (C.M.-V.); 2 Nursing Research, Innovation and Development Centre of Lisbon (CIDNUR), Nursing School of Lisbon, 1600-190 Lisbon, Portugal; rgarcf@unileon.es; 3 SALBIS Research Group, Department of Nursing and Physiotherapy, Faculty of Health Sciences, Campus de Ponferrada, Universidad de León, 24401 León, Spain; equis@unileon.es; 4 Nursing School, University of Minho, 4710-057 Braga, Portugal; fmartins@ese.uminho.pt; 5 Nursing Research Center (CiEnf), Health Sciences Research Unit: Nursing (UICISA: E), School of Nursing of Coimbra (ESEnfC), 3004-011 Coimbra, Portugal

**Keywords:** lymphedema, breast neoplasms, physical exercise, prevention, thermography, oncology rehabilitation, therapy

## Abstract

**Background/Objectives:** Breast cancer-related lymphedema (BCRL) is a chronic disease with lasting effects, making it one of the most feared sequelae of breast cancer with significant personal and social impacts. Therapeutic exercises play a fundamental role in its treatment. This systematic review aims to provide the most up-to-date findings on the impact of physical exercise on the management of BCRL. **Methods:** Following the PRISMA statement guidelines, searches were conducted in the Web of Science, Scopus, and Science Direct databases. **Results:** Sixteen studies published between 2019 and 2024 were analyzed in detail. The combination of strength and aerobic exercises emerged as an effective strategy for both the treatment and prevention of lymphedema, also highlighting the innovative potential of virtual reality. **Conclusions:** It is essential to emphasize tailoring exercise programs to each patient individually. Additionally, the promising role of thermography as a non-invasive and safe tool for evaluating lymphedema progress is underscored.

## Simple Summary

The findings presented in this article provide compelling evidence supporting the synergistic benefits of combining strength and aerobic exercises as a highly effective and scientifically robust strategy for managing lymphedema. Furthermore, the research underscores the innovative potential of thermography, emphasizing its role as a safe, sustainable, non-invasive, and highly effective tool for assessing the progress of therapeutic interventions. This integrated approach not only offers a comprehensive framework for evaluating and enhancing treatment efficacy but also holds significant promise for advancing patient care in this field.

## 1. Introduction

Breast cancer is the most prevalent tumor among women globally [1]. Breast cancer-related lymphedema (BCRL) is a debilitating complication of cancer treatment, especially after axillary lymph node dissection and radiotherapy. The recent research suggests that axillary lymph node dissection (ALND) may be safely omitted in patients with breast cancer undergoing mastectomy who present with one–two positive sentinel lymph nodes. This approach provides a less invasive alternative with comparable oncological outcomes [2]. However, while initial findings are promising, further randomized clinical trials are necessary to confirm these results and establish clearer, more definitive treatment guidelines for patients. BCRL affects more than one in six breast cancer survivors [3], causing progressive swelling of the arm or hand and profoundly affecting their physical and psychosocial quality of life [4,5].

Although there is no universal consensus on the criteria defining lymphedema, it is generally diagnosed when there is a difference of at least 2 cm in the arm circumference or 200 mL in the volume between the limbs [6]. Patients with lymphedema experience a significant decrease in their physical and mental health [7], affecting their body image, appearance, sexuality, and social life [8]. This is driving the research on preventive and treatment strategies.

The treatment of BCRL focuses on reducing excess arm volume and managing symptoms such as heaviness and swelling [9]. Therapeutic options include compression therapy, therapeutic exercises, pharmacotherapy, and complex decongestive physiotherapy [10], encompassing various techniques to improve lymphatic flow [11,12,13].

In this regard, therapeutic exercises are essential in treating lymphedema, as they improve lymphatic flow through the contraction and relaxation of the arm, shoulder girdle, and trunk muscles [14]. Types of exercise include aerobic, strength, and flexibility or mobility exercises, which promote a healthy lifestyle [15] and are considered safe without increasing the risk of developing lymphedema after breast cancer treatment [16].

Given the limitation of medical resources globally, it is crucial to develop an effective preventive strategy to address this complication. Although numerous reviews on the effects of exercise in women with breast cancer exist, these have limitations that hinder their generalization [17,18,19]. Therefore, a more specific understanding of the effects of all types of exercise on BCRL is required [15]. Consequently, this systematic review provides the most recent findings on the impact of physical exercise on BCRL from 2019 to 2024.

## 2. Materials and Methods

### 2.1. Data Sources and Searches

A systematic literature review was conducted between November 2023 and February 2024, following the Preferred Reporting Items for Systematic Reviews and Meta-Analyses (PRISMA) guidelines [20]. Studies published from 2019 to 2024 were included in searches in three databases: Web of Science (WOS), Scopus, and Science Direct.

Search terms related to breast cancer, lymphedema, and thermography were used. Search equations included combinations of keywords such as “thermography”, “gym”, “training”, “rehabilitation”, and “virtual reality”, along with specific terms for breast cancer and lymphedema. Additionally, manual searches were conducted in the references of the retrieved articles to identify additional relevant citations.

### 2.2. Inclusion and Exclusion Criteria

The selection of studies was based on predefined inclusion and exclusion criteria according to the PICO concept: (1) population: breast cancer patients diagnosed with or at risk of developing BCRL; (2) intervention: studies incorporating weight training, aerobic exercise, active exercises, at-home exercises, or virtual reality; (3) control: studies with a control group or at least two comparative groups; (4) outcomes: studies evaluating BCRL, the L-Dex index, CLUE, direct arm volume measurement, and water displacement measurement, evaluating the volume differences between arms; (5) study type: empirical studies; (6) language: articles in English, Spanish, and Portuguese. Articles not fully accessible, published before 2019, or not meeting the inclusion criteria were excluded.

### 2.3. Data Extraction and Synthesis

Data from each study were extracted using a literature review form designed by the authors.

### 2.4. Quality Assessment

Two researchers assessed the risk of bias in each study using the Cochrane ROB 2 tool [21]. Any discrepancies were resolved with the intervention of a third evaluator.

## 3. Results

**Figure 1:**
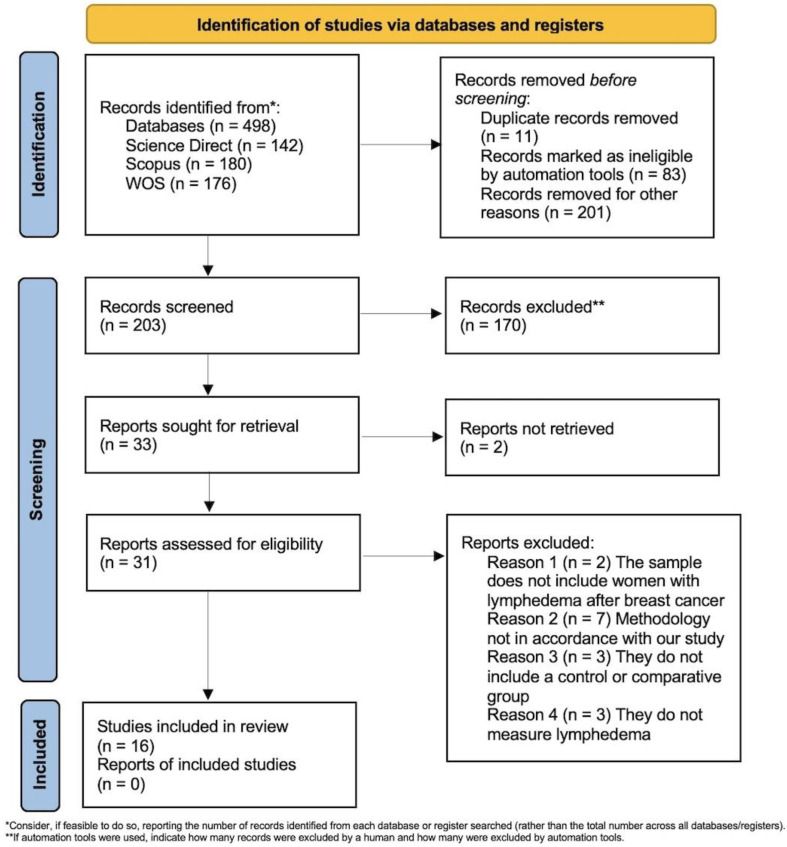
PRISMA 2020 flow diagram for new systematic reviews, which included searches of databases and registers only. Source [20]. This work is licensed under CC BY 4.0. to view a copy of this license, visit https://creativecommons.org/license/by/4.0/ (accessed on 15 December 2024).

From the initial 498 articles obtained, 203 were selected after checking for duplicates and applying the inclusion and exclusion criteria. After reviewing the titles and abstracts, 170 were excluded. Of the remaining 33, detailed information was retrieved from 31, with 2 cases where it was impossible. After a thorough review, 15 articles were excluded for various reasons, leaving 16 for the final review (Figure 1). Data were extracted and synthesized into a summary table (Table 1).

### 3.1. General Characteristics of the Included Studies

Of the 16 selected studies, all of them clinical trials, 4 were randomized controlled pilot studies [22,32,34,36], 1 was a single-center clinical trial [23], 2 were multicenter clinical trials [25,33], 1 was a quasi-randomized trial [24], and 8 were randomized clinical trials [26,27,28,29,31,35,37,38]. Of these selected studies, two focused on the feasibility of using thermography during exercise programs and in the diagnosis of lymphedema in mastectomized women [36,37], while the others investigated how physical exercise influences the prevention or treatment to improve lymphedema [22,23,24,25,26,27,28,29,31,32,33,34,35,38].

### 3.2. Location Information

The included randomized clinical trials came from various parts of the world. They were identified as follows: (1) three studies in Asia: one from Japan [22] and two from China [28,31]; (2) seven in Europe: two from Spain [23,25], three from Denmark [26,27,33], one from Italy [34], and one from Poland [36]; (3) two in North America: one from New York [38] and another from Philadelphia [29]; (4) one in South America, conducted in Brazil [37]; (5) one in the Middle East, in Iran [32]; and (6) two in Africa, both in Egypt [24,35].

### 3.3. Participant Information

The sample consisted of 1358 women; 855 were assigned to the intervention group and 503 to the control group. Women diagnosed with axillary web syndrome [23,36], those at risk of developing lymphedema [26,27,28,31,33,37], as well as those already suffering from this condition [22,24,25,29,32,34,35,38] were included.

### 3.4. Risk of Bias (ROB) of the Included Studies

**Figure 2:**
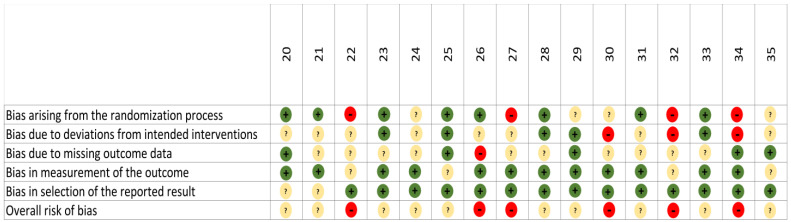
ROB of the included studies. 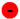 Hig risk 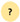 Some concerns 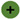 Low risk.

None of the 16 evaluated studies demonstrated a low risk of bias (Figure 2). Of these, 10 showed a moderate risk, mainly due to missing data [25,35,38], unclear interventions [23,26,33], suboptimal randomization processes [31], imprecise outcome measurements [27,37], and issues with interventions and outcome selection [22]. The remaining six presented a high ROB [24,28,29,32,34,36].

### 3.5. Primary Outcome Measures

In the review, all 16 articles examined variations in arm volumes. Most (13 out of 16) used a measurement method. The methods employed included water displacement [32,33], limb circumference [23,24,25,28,31,35], perometry [29,38], and electrical impedance measurement using L-Dex [26,27]. Additionally, one study used a thermal camera to measure the arm volume [37], while three others combined two methods [22,34,36].

### 3.6. Effects of the Interventions

Among the 16 reviewed studies, most intervention programs lasted 8 to 12 weeks, the predominant duration in 6 studies. However, two studies extended their programs to 52 weeks, while others varied between 3 and 48 weeks.

A variety of approaches were found regarding exercise modalities. Some studies implemented strength exercises [29,32,33,37], another focused on aerobic exercise [26], and another combined both types [27]. Some researchers opted for mobility exercises [22,23,25,28,34]. Additionally, studies using virtual reality exercises [24,30,31,35] were found.

- Strength Exercise Interventions

Most studies investigated strength exercises combined with supervised sessions with self-administered home exercises [29,32,33]. These interventions included warm-ups, stretching, and exercises with dumbbells, shin guards, and elastic bands. Schmitz et al. [29] added compression garments but did not find significant changes in the arm volume.

In contrast, Das Virgens Aquino et al. [37] focused on individual exercises thrice a week for 20 weeks, including stretching, kinesiology, strength training, and relaxation. This study showed a significantly lower temperature in the plastron area compared to the control breast.

- Aerobic Exercise Interventions

Bloomquist et al. [26] conducted a 52-week study on “football fitness” training twice a week. These training sessions included a warm-up, specific football exercises, and small-team games outdoors. At 6 months, significant differences were found in the arm volumes of the intervention group compared to those of the control group.

- Combined Strength and Aerobic Exercise Interventions

Bloomquist et al. [27] conducted a unique study combining strength and aerobic exercises. Over 12 weeks, they divided participants into two groups: high-intensity and low-intensity groups. The high-intensity group performed low- and high-intensity exercises during the first 6 weeks, followed by multidisciplinary exercises with high-intensity and medium–high aerobic activity in the next 6 weeks. The low-intensity group participated in walking programs. Both groups received health education (HE). Changes in the arm volume differences were observed, with a significant benefit in the high-intensity group.

- Gentle Mobility Exercise Interventions

Torres-Lacomba et al. [23] employed 45 min sessions with arm mobility exercises, functional activities, and home exercises. Muñoz-Alcaraz et al. [25] combined health education, neurodynamic activities, and five individualized daily activities. Both groups received complementary treatment, including manual lymphatic drainage and compression garments, showing significant differences in the arm volume in the intervention group.

In contrast, Arinaga et al., Zhu et al., and Carretti et al. [22,28,34] focused on mobility and self-care exercises. Carretti et al. [34] also incorporated home sessions twice weekly but found no significant differences in the arm volume over time.

Torres-Lacomba et al. [23] also evaluated shoulder disability with the Oxford Shoulder Scale (OSS), showing improvements in the intervention group. Carretti et al. [34] used the ULL-23 questionnaire to assess quality of life, observing a significant improvement in the intervention group. Finally, Muñoz-Alcaraz et al. and Zhu et al. [25,28] evaluated arm, shoulder, and hand disability with the DASH scale, finding better scores in the intervention group.

- Virtual Reality Exercise Interventions

Regarding virtual reality exercise interventions, Atef et al. and Basha et al. [24,35] used Nintendo Wii and Xbox Kinect in virtual reality programs with games like darts and tennis, as well as pneumatic compression techniques and manual lymphatic drainage. Basha et al. [35] also added strength exercises with dumbbells. Both studies found significant differences in the arm volumes and improvements in the upper limb function according to the QuickDASH-9 scale.

Fu et al. and Du et al. [30,31] implemented the optimal lymph flow (TOLF) intervention, providing lymphatic and limb mobility exercises through a web and mobile platform. They observed a reduction in the proportion of patients with limb volume differences in the TOLF group and improvements in symptoms associated with lymphedema according to the BCLE-SEI.

These findings suggest that no evaluated exercise triggered or exacerbated lymphedema in patients at risk of BCRL.

## 4. Discussion

Based on the 16 articles selected for this review, various exercise interventions, including strength training, aerobic training, combined strength and aerobic training, virtual reality, and mobility exercises, are effective at managing BCRL in patients at risk and those with the condition already developed.

### 4.1. Strength and Combined Exercises

Interventions based solely on strength exercises, whether supervised or self-administered at home, did not produce significant changes in arm volumes. However, the combination of strength and aerobic exercises, as observed in Bloomquist et al. [27], demonstrated significant benefits, especially in the high-intensity group. This suggests that combining different types of exercise may be more effective than individual approaches at managing the risk and treatment of lymphedema. These findings support the recommendations of the National Lymphedema Network, which indicates that individuals affected by lymphedema or at risk of developing it can benefit from the safe practice of a combination of aerobic and strength exercises [39].

### 4.2. Aerobic Exercises

In the study by Bloomquist et al. [26], which implemented a “football fitness” training program, positive results were observed in reducing the risk of lymphedema, highlighting the importance of including aerobic exercises in exercise programs for patients at risk of lymphedema. However, this study lasted 52 weeks, and significant changes were observed until week 24. This prolonged duration raises questions about the need and effectiveness of shorter interventions. Furthermore, caution is essential in interpreting these results based on a single randomized clinical trial with a small sample size. Therefore, more rigorous studies with more participants are required to provide a clearer understanding and generalizable conclusions.

### 4.3. Mobility Exercises

Studies on mobility exercises, virtual reality, and self-care have shown benefits in managing the risk of lymphedema. This suggests that less intensive interventions, such as gentle exercises or virtual reality games, can be effective and well tolerated by patients. These observations are supported by previous research [40], which found that a low-intensity home exercise program combined with self-care improved the quality of life and arm function in women with BCRL. Recent studies [41,42] have developed virtual reality rehabilitation systems, concluding that they are feasible and easy to learn for breast cancer patients. Some studies also incorporated manual lymphatic drainage or compression garments, which may have influenced the results.

Overall, the findings suggest that various exercise interventions, including strength, aerobic, mobility, virtual reality, and app-based technology exercises, can be beneficial for managing the risk of lymphedema in breast cancer patients. However, it is crucial to personalize and adapt the exercise to individual needs [43]. Additionally, thermography stands out as a recommended option for measuring lymphedema, being a non-invasive and safe technique [44,45]. However, more research is needed to understand the long-term effects of these interventions on lymphedema prevention and management.

#### Limitations

The limitations of this systematic review are important to acknowledge. One key limitation is the reliance on studies with small, predominantly female samples, which may affect the generalizability and validity of the results. The scarcity of studies with larger and more diverse populations, particularly those including men, posed a significant challenge. Additionally, the heterogeneity in the interventions, sample sizes, and study durations further complicated the synthesis of clear conclusions. The small sample sizes limit the power and applicability of the findings, restricting their broader use in diverse populations. The variation in study designs also hindered consistent and comparable conclusions, and many studies had short follow-up periods, which complicates the interpretation of long-term effects and sustainability. The limited duration and variability in the results also contributed to the complexity of drawing definitive conclusions.

Therefore, we strongly emphasize the need for longitudinal studies to better evaluate these effects over time. In the interim, caution should be exercised when generalizing the findings, and recommendations should be personalized to the specific context and characteristics of each population. Additionally, selection and reporting biases were identified in the studies reviewed. Selection biases may have influenced the representativeness of the samples, and information biases may have led to the preferential publication of studies with positive results, introducing a bias toward more optimistic conclusions.

These limitations underscore the need to interpret the findings cautiously and advocate for more rigorous standards in the design and publication of future research in this area. Given that this is a systematic review, no definitive conclusions can be made regarding the absolute effects of exercise interventions on lymphedema. Further research is necessary to achieve a more comprehensive understanding of the benefits and limitations of these interventions.

## 5. Conclusions

This systematic review concludes that the combination of strength and aerobic exercises offers significant benefits in treating and preventing lymphedema, being more effective than individual approaches. It is crucial to adapt these programs to each patient’s specific needs. Additionally, thermography is a promising tool for evaluating lymphedema, as it is non-invasive, painless, and safe, providing valuable information on the effectiveness of exercise interventions.

## Figures and Tables

**Table 1:** Results of the systematic review.

Study	Sample Design	Intervention	Variables	Results
Arinaga et al., (2019) [22]	Randomized controlled pilot study. Women with BCRL. GI: n = 22; GC: n = 21.	GI: daily self-care program, 6 min for 6 months with Radio Taiso, arm exercises, DL, informational booklet. GC: informational booklet. Measurements: 0, 1, 2, and 6 months.	L-Dex; REV	L-Dex showed no significant changes between groups throughout the study. REV showed a significant interaction between group and time.
Torres-Lacomba et al., (2022) [23]	Randomized clinical trial. Women with ARM syndrome post-breast cancer. GI: n = 48; GC: n = 48.	3-week intervention with 3 weekly visits, including education. GI: 45 min sessions (30 min for GC) with DL, arm exercises, and functional activity. GC: standard home exercises. Measurements: 0, 3 weeks, and 3 and 6 months.	Arm circumference; AROM; self-reported OSS	Circumference improved in both groups throughout follow-up, with greater improvement in GI in all measurements. AROM and OSS improved significantly in GI in the 0 and 3 measurements compared to GC.
Atef et al., (2020) [24]	Quasi-randomized clinical trial. Women with UPML. VR group: n = 15; PNF group: n = 15.	VR group: joint warm-up + 30 min exercise with Nintendo Wii. Two sessions/week for 4 weeks. PNF group: controlled arm movements 20 min for 4 weeks + home exercises + EpS. Measurements: 0- and 4-weeks post-intervention.	Circumferential method (EAV); QuickDASH-9	There were significant differences in EAV and QuickDASH-9 between the VR and PNF groups before and after intervention. The VR group showed greater improvement in lymphedema status and upper extremity function compared to the PNF group.
Muñoz-Alcaraz et al., (2022) [25]	Randomized multicenter controlled trial. Women with BCRL. GI: n = 32; GC: n = 32.	GI: TAPA treatment (EpS + neurodynamic activity for ADLs + proprioceptive facilitation exercises + low compression bandaging). 10 sessions × 30 min, 2 times per week. GC: preventive measures + exercise + compression garments for 5 weeks + 10 sessions of 60 min of CDT 3 times a week. Measurements: 0 and 3 months.	Circumference; goniometer; QuickDASH-9	Significant differences in % reduction of edema volume and significant improvements in joint balance in GI compared to baseline.
Bloomquist et al., (2021) [26]	Randomized controlled trial. Women at risk of BCRL. FFG group: n = 46; GC: n = 22.	FFG group: fitness football training 2 times per week, 52 weeks, including warm-up, football exercises, and small team games. GC: no physical activity restrictions. Measurements: 0, 6, and 12 months.	L-Dex; EORTC QLQ BR23 version 3.0; QuickDASH-9	There was a significant reduction in L-Dex in FFG at 6 months, but no significant changes in GC. At 12 months, there were no intergroup differences, and there were no differences in other outcomes.
Bloomquist et al., (2019) [27]	Randomized trial. Women at risk of BCRL. HIGH group: n = 64; LOW group: n = 66.	HIGH group: 12 weeks of supervised group exercise. Low- and high-intensity aerobic and all-sport exercises. LOW group: individualized walks + promotion of physical activity in ADLs. Both groups: EpS. Measurements: 0, 6, 12, and 39 weeks.	L-Dex; self-reported swelling; self-reported BCRL symptoms	After the intervention, equivalence between groups in L-Dex, swelling symptoms, and heaviness were observed. Clinically relevant reductions in breast and arm symptoms were observed in the HIGH group.
Zhu et al., (2023) [28]	Randomized trial. Women at risk of BCRL. GI: n = 60; GC: n = 60.	GI: mirror therapy from the first day post-intervention. Free movement of the upper limb 2–3 min × 3 times. GC: handball exercises + arm raises, two daily sessions × 4 days a week. Measurements: 0, 2, 4, and 8 weeks.	Circumferential method; QuickDASH; Constant–Murley Score (CMS)	After the intervention, patients in the GI had higher CMS scores and lower QuickDASH scores compared to GC patients. No significant differences in bilateral circumference between groups.
Schmitz et al., (2019) [29]	Randomized clinical trial. Women with BCRL. GI with exercise: n = 87; GC: n = 90; weight loss group: n = 87.	Both groups: initial compression garment. GI: home exercises + phone follow-up + monthly classes with warm-up, stretching, and resistance exercises × 52 weeks. Weight loss group: 24 weekly sessions with dietitian. Combined group: initial exercise and weight loss. Measurements: 0 and 12 months.	Perometry	No differences between groups, either initially or at 12 months, in percentage or absolute differences between limbs.
Fu et al., (2022) [30]	Randomized clinical trial. Women with BCRL. GI: n = 60; GC: n = 60.	GI: TOLF mHealth platform for web and mobile. Content: lymphedema + self-care + exercises and diagnosis + instructional videos. GC: access to the ARM Precaution web and mobile program upon first visit. Measurements: 0- and 12-weeks post-intervention.	Infrared Perimeter; pain and lymphedema symptoms; lymphedema and breast cancer symptom experience index	GI: fewer patients reported swelling and heaviness, and there was a difference in limb volumes compared to GC. For pain, median scores were lower in GI compared to GC.
Du et al., (2022) [31]	Randomized clinical trial. Women at risk of BCRL. GI: n = 46; GC: n = 46.	GI: TOLF with full access to web and mobile platforms (educational modules on lymphedema, self-care, and eight therapeutic exercise videos). GC: access to the same educational module and four TOLF mHealth therapeutic exercise videos. Intervention duration: 3 months. Measurements: 0- and 3-months post-intervention.	Circumferential method; BCLE-SEI	There were significant improvements in BCLE-SEI in GI compared to GC, both in the number and severity of symptoms. Both groups showed improvements in all study outcomes during the three months.
Pasyar et al., (2019) [32]	Controlled pilot study. Women with BCRL. GI: n = 20; GC: n = 20.	GI: yoga + standard lymphedema care (two sessions/week + one at home with DVD). GC: standard lymphedema care. Duration: 8 weeks. Measurements: 0, 4, and 8 weeks.	Water displacement volume meter	There were no significant differences in edema volume between both groups in the fourth and eighth week after intervention.
Ammitzbøll et al., (2019) [33]	Randomized multicenter controlled trial. Women at risk of BCRL. GI: n = 82; GC: n = 76.	GI: Phase 1:20 weeks of supervised exercise + home exercise 3×/week. Phase 2:30 weeks of home exercise 3×/week. GC: usual care without specific intervention. Measurements: 0 and 12 months.	Water displacement: DXA; heaviness, tightness, and swelling symptoms (NRS)	There were no significant differences between groups in the arm volume or the incidence of lymphedema. Adjusted analyses revealed no differences between groups in the NRS score for swelling, tightness, or heaviness.
Carretti et al., (2022) [34]	Monocentric pilot study. Women with BCRL. GI: n = 16; GC: n = 14.	GI: APA protocol with exercise specialist. A total of 15 group sessions of 50 min and 2 days/week at home (roll-up). Hand-walk exercise for wrist, hand, and finger proprioception. GC: passive therapy with acupuncture.	Circumferential method; upper limb lymphedema 27 scale (ULL-27)	ULL27 results indicated a significant improvement in GI’s overall quality of life. Participants reported better upper extremity functionality and reduced edema. Ultrasound and volumetric measurements remained practically unchanged, but wrist mobility, pain perception, and quality of life improved significantly in GI.
Basha et al., (2022) [35]	Single-blind randomized trial. Women with BCRL. Xbox group: n = 30; Resistance exercise group: n = 30.	Both groups: DL, exercises, and skin care. Xbox Kinect group: VR games (darts, bowling, boxing…) five sessions/week × 8 weeks. Resistance exercise group: stretches, dumbbell exercises. Measurements: 0- and 8-weeks post-intervention.	VAS scale; QuickDASH; digital goniometer (shoulder ROM); hand dynamometer; circumferential method (ELV)	There were significant differences in favor of the Xbox group in VAS, DASH, and ROM. The resistance exercise group had improved shoulder flexion strength, external rotation, abduction, and hand grip.
Dȩbiec-Bąk et al., (2020) [36]	Pilot study. Women at risk of BCRL. GI: n = 20; GC: n = 23.	Objective: to evaluate the utility of thermography in lymphedema diagnosis.	Circumferential method; use of Limb Volumes Professional 5.0 software; Thermo Vision Flir Systems 2.9 thermal camera	In the control group, the difference in volumes between the operated and contralateral limbs was 3.6%, with a higher temperature on the non-operated side. In the test group, the size difference was 29.3%, showing an inverse trend in cases of mild or moderate edema. A negative Spearman correlation coefficient (−0.34) was also observed between the lymphedema size and surface temperature.
das Virgens Aquino et al., (2022b) [37]	Randomized clinical trial. Women at risk of BCRL. n = 20.	Objective: to evaluate the feasibility of using thermography during a physical rehabilitation program in mastectomized patients. Participants: individual exercise program three times/week × 20 sessions. Stretching, kinesiotherapy, and resistance exercises with dumbbells and ankle weights. Measurements: 0, 10, and 20 weeks.	Flir Systems T420 thermal camera	No significant differences were found in the plastron area thermograms between sessions in the breast region thermography. However, a significant reduction in temperature was observed in the operated region compared to the control breast in the first and tenth sessions.

Abbreviations: ADLs: activities of daily living; ARM: axillary web syndrome; BCLE-SEI: breast cancer lymphedema severity experience index; BCRL: breast cancer-related lymphedema; CDT: complex decongestive therapy; DL: lymphatic drainage; ELV: excess limb volume; EpS: health education; EESS: upper extremities; FFG: football fitness group; GC: control group; GI: intervention group; NRS: Numeric Rating Scale for Symptoms; PNF: proprioceptive neuromuscular facilitation group; REV: relative edema volume; TAPA: proprioceptive anti-edema therapy focused on activity; TOLF: the optimal lymph flow; UPML: unilateral post-mastectomy lymphedema patient; VR: virtual reality.

## Data Availability

Data are contained within the article.
